# Remote cerebellar hemorrhage following thoracic spinal surgery of an intradural extramedullary tumor: a case report

**DOI:** 10.1186/s13256-015-0541-8

**Published:** 2015-03-26

**Authors:** Masazumi Suzuki, Takashi Kobayashi, Naohisa Miyakoshi, Eiji Abe, Toshiki Abe, Yoichi Shimada

**Affiliations:** 1 Department of Orthopedic Surgery, Akita Kousei Medical Center, 1-1-1 Iijima, Nishifukuro, Akita 011-0948 Japan; 2 Department of Orthopedic Surgery, Akita University Graduate School of Medicine, 1-1-1 Hondo, Akita, 010-8543 Japan

**Keywords:** Postoperative complication, Intradural extramedullary tumor, Remote cerebellar hemorrhage, Spinal surgery, Thoracic spine, Neurological signs

## Abstract

**Introduction:**

Remote cerebellar hemorrhage is a rare complication of spinal surgery. Although loss of cerebrospinal fluid seems to play an important role in the pathogenesis of this complication, the detailed mechanism of remote cerebellar hemorrhage after spinal surgery remains unclear. We report the case of a patient with remote cerebellar hemorrhage following thoracic spinal surgery of an intradural extramedullary tumor and discuss this entity with reference to the literature.

**Case presentation:**

A 57-year-old Japanese woman presented to our hospital with back pain, dysuria, and numbness of both legs. A neurological examination was performed, and imaging was performed with ordinary radiography, magnetic resonance imaging, and computed tomography. Her magnetic resonance imaging scan showed an intradural extramedullary tumor at the T3 level. A tumor resection and T1-T5 pedicle screw fixation were performed. Twelve hours after spinal surgery, she complained of unexpected dizziness, nausea, and vomiting. A total of 850mL of serosanguineous fluid had been drained at that time, and drainage was stopped. An urgent brain computed tomography scan showed a cerebellar hemorrhage. She was treated conservatively, and was able to leave hospital six weeks after the initial operation, without any neurological deficits except for slight ataxia.

**Conclusions:**

Remote cerebellar hemorrhage has to be suspected when unexpected neurological signs occur after spinal surgery. If an excessive amount of cerebrospinal fluid drains from the drainage tube after spinal surgery, drainage should be stopped.

## Introduction

Remote cerebellar hemorrhage (RCH) following spinal surgery is a rare complication [1-25]. Although loss of cerebrospinal fluid (CSF) plays an important role in the pathogenesis of this complication [1-25], the detailed mechanism of RCH after spinal surgery remains unclear. Here, we present a case of RCH after thoracic spinal surgery for an intradural extramedullary tumor, along with a review of previously reported cases and a discussion of the mechanism of RCH.

## Case presentation

A 57-year-old Japanese woman, with no past medical history, presented to our institution with a one-year history of abdominal pain, a two-month history of back pain, numbness of both her legs, and a one-month history of dysuria. She initially reported abdominal pain and underwent extensive gastroenterological evaluation at another hospital, including an esophagogastroduodenoscopy, which was unremarkable.

**Figure 1:**
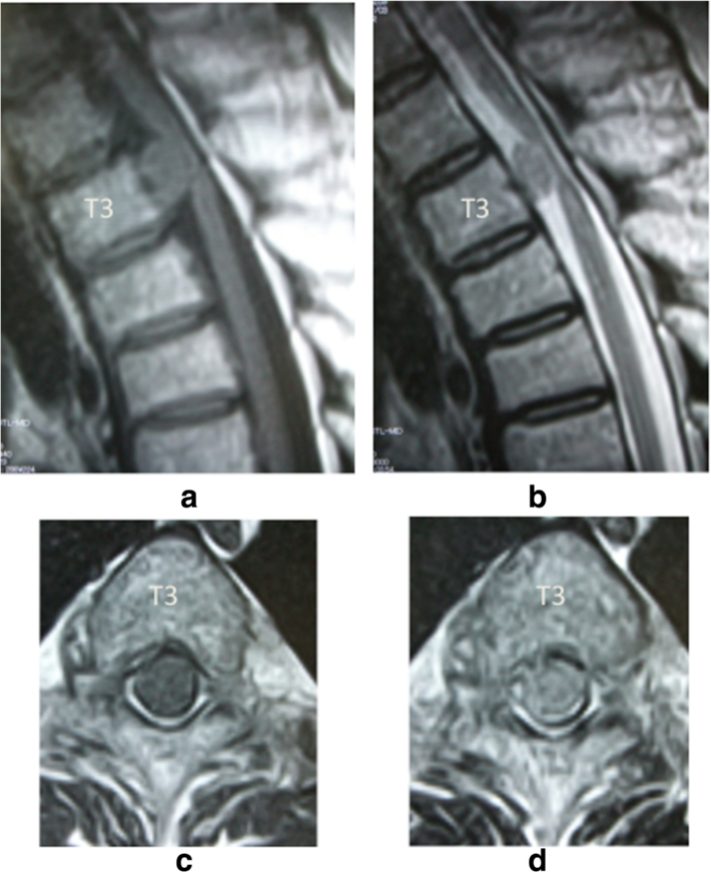
**Preoperative magnetic resonance images.** Sagittal T1-weighted **(a)** and T2-weighted **(b)** magnetic resonance images of the thoracic spine, demonstrating an intradural extramedullary mass anterior to the spinal cord at the T3 level. The mass was iso-intense on T1-weighted imaging and T2-weighted imaging. Axial T1-weighted **(c)** and T2-weighted **(d)** magnetic resonance images show that the tumor seemed to be completely covered by the spinal cord.

Her physical examination revealed no motor weakness and normal tendon reflexes. She felt hypoesthesia below the umbilicus. Magnetic resonance imaging (MRI) results demonstrated a large intradural extramedullary mass at the T3 level that was compressing her spinal cord from the ventral side (Figure 1).

**Figure 2:**
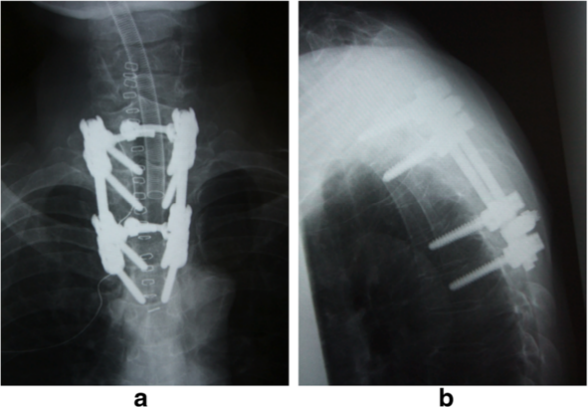
**Postoperative radiographs.** Postoperative anterior-posterior **(a)** and lateral **(b)** radiographs showing T1-T5 pedicle screw instrumentation.

The intradural extramedullary tumor was resected through a laminectomy of T2-T4 and a facetectomy of T2-T3 and T3-T4 in the prone position under transcranial motor-evoked potential (MEP) monitoring. As the tumor was completely covered by her spinal cord, it was surgically removed by rotation of the spinal cord using tenting of the dentate ligament. After tumor resection, the dura that adhered to the tumor was cauterized. A watertight repair of the dura was performed, using fibrin glue to avoid CSF leakage. A T1-T5 pedicle screw fixation was performed (Figure 2). Abnormal MEP signals were observed on her left leg during and after the tumor resection. A subfascial drain was put in place, with negative pressure. After she woke the motor power was weakened to grade three to four in her left knee and ankle. The total operating time was 4 hours 39 minutes, and the amount of bleeding was 108g. The histological diagnosis of the tumor was a meningioma.

**Figure 3:**
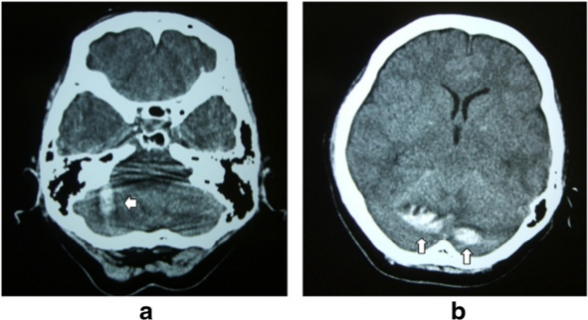
**Non-enhanced plain computed tomography scan of the head taken 13 hours after spinal surgery, demonstrating an acute cerebellar hemorrhage in the superior folia of the cerebellar hemispheres (white arrows). (a)** At the low cerebellum level, **(b)** At the cerebral peduncles level.

**Figure 4:**
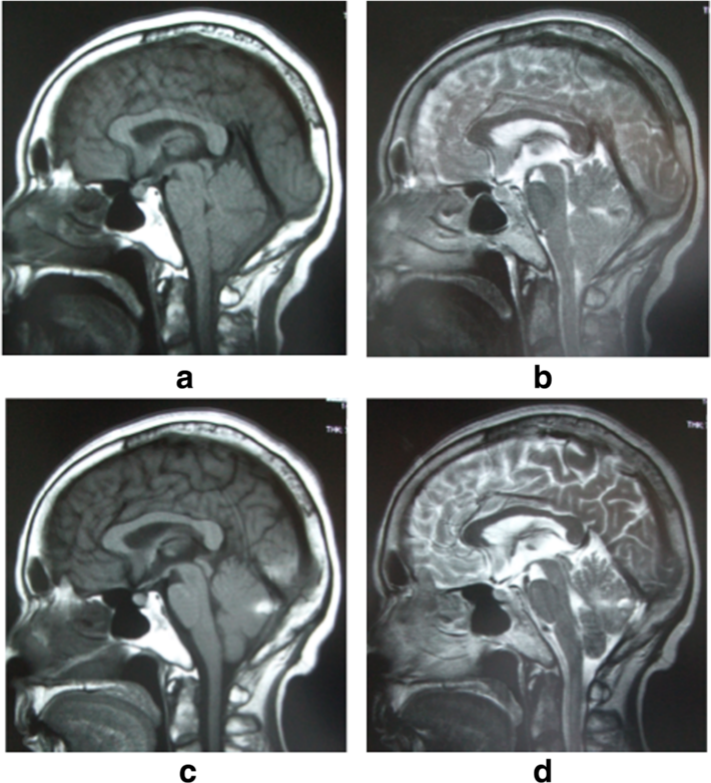
**Sagittal magnetic resonance imaging taken 13 hours and 12 days after spinal surgery.** Sagittal T1-weighted **(a)** and T2-weighted **(b)** magnetic resonance images taken 13 hours after spinal surgery, demonstrating herniation of the cerebellar tonsils. Sagittal T1-weighted **(c)** and T2-weighted **(d)** magnetic resonance images taken 12 days after spinal surgery demonstrating ascent of the cerebellum to the normal position.

Twelve hours after surgery, she developed nausea and confusion, and her clinical status deteriorated with loss of consciousness (Glasgow Coma Scale score of seven). A total of 850mL serosanguineous fluid had been drained at that time, and drainage was stopped. An emergency brain computed tomography (CT) scan demonstrated an acute cerebellar hemorrhage in the superior folia of the cerebellar hemispheres (Figure 3). An MRI scan demonstrated a herniation of the cerebellar tonsils (Figure 4a, b). She was treated conservatively with anti-edema and antihypertensive drugs, and her clinical status improved gradually. After removal of the drain, there was no CSF leakage. The results of her follow-up CT scan performed one week later showed that her hematoma and brain edema were decreased. Twelve days later, the results of her follow-up MRI scan showed ascent of the cerebellum to the normal position (Figure 4c, d). At six weeks after surgery, she had slight ataxia and was discharged with a cane. At her one-year follow-up assessment, she had a normal neurological examination except for hypoesthesia of the right leg, and there was no CSF collection visible on her MRI scan.

## Discussion

Our case report has two characteristics. First, this case of thoracic meningioma that was located anterior to the spinal cord presented with a one-year history of undiagnosed abdominal pain. Lyons *et al*. [26] reported a similar case presenting as chronic abdominal pain. Second, although the tumor (which completely covered the spinal cord) was totally removed with a posterior surgical approach, our patient had some left lower extremity weakness postoperatively that improved gradually. A total resection of intradural extramedullary tumors located anterior to the spinal cord can be performed using an isolated posterior approach, with rotation of the spinal cord and tenting of the dentate ligament [27,28].

RCHs are rare and dramatic complications can follow spinal surgery. Prevention is important, because RCHs sometimes follow a fatal course. Sporadic cases have been published since the first description by Chadduck [4]. At the time of writing, 32 cases of RCH after spinal surgery have been reported in the English-language literature (Table 1). Including the present case, the 33 cases consisted of 23 women and 10 men, with an age range of 36 to 85 years (mean: 60.9 years). Initial surgery was performed at the lumbar spine in 21 cases, thoracic spine in six, cervical spine in five, and thoracolumbar spine in one. A dural tear during surgery was present in 26 cases, but was not noticed in seven cases. The neurological symptoms were detected between 0 and 192 hours (mean: 45.7 hours) after surgery. A total of 16 RCHs were resolved with conservative treatment, but three patients died or developed serious paresis [1-3,5,8,10,11,15,19,21-23]. However, in severe cases, emergency surgical intervention with ventricular drainage or posterior fossa craniotomy was needed. Cranial surgery was performed in 14 patients, nine of whom improved, and five died or had serious paresis after surgery [4,6,7,9,11-14,17,18,20,24,25].

**Table 1:** **Clinical parameters and outcomes in previous reports of remote cerebellar hemorrhage**

**Author (year)**	**Surgery**	**Location**	**Age, sex**	**Onset**	**Dural tear**	**Treatment**	**Results**
Chadduck (1981) [4]	laminectomy	CS	59, M	2 days	present	surgery	improved
Mikawa *et al*. (1994) [17]	C1/2 fusion, durotomy	CS	75, M	1 day	present	surgery	died
Andrews and Koci (1995) [1]	scoliosis correction	LS	36, M	36 hours	unknown	conservative	quadriparesis
Friedman *et al*. (2002) [8]	posterior thoracic disc herniation removal	TS	43, M	12 hours	present	conservative	improved
	PSF	LS	56, F	2 days	present	conservative	improved
Thomas *et al*. (2002) [22]	IETR	TLS	38, F	5 days	present	conservative	improved
Farag *et al*. (2005) [7]	PSF	LS	43, F	36 hours	present	surgery	improved
Karaeminogullari *et al*. (2005) [12]	PSF	LS	73, F	2 days	present	surgery	improved
Nakazawa *et al*. (2005) [19]	IETR	CS	74, F	perioperative	present	conservative	improved
Konya *et al*. (2006) [15]	PSF	LS	48, F	12 hours	present	conservative	improved
Calisaneller *et al*. (2007) [2]	PSF	LS	67, F	8 days	present	conservative	improved
Cornips *et al*. (2007) [5]	thoracoscopic microdiscectomy	TS	48, F	3 days	unknown	conservative	died
Hashidate *et al*. (2008) [9]	vertebral tumor resection	TS	85, F	40 hours	unknown	surgery	improved
Cevik *et al*. (2009) [3]	laminectomy	LS	79, F	3 days	unknown	conservative	improved
	PSF	LS	68, F	7 days	unknown	conservative	improved
Enel *et al*. (2009) [6]	PSF	LS	51, F	30 hours	present	surgery	died
Khong and Jerry Day (2009) [14]	PSF	LS	70, F	36 hours	present	surgery	improved
Morofuji *et al*. (2009) [18]	laminectomy	TS	51, M	18 hours	present	surgery	improved
Pallud *et al*. (2009) [20]	laminectomy	LS	73, F	3 days	present	surgery	improved
Ulivieri *et al*. (2009) [23]	microdiscectomy	LS	53, M	2 hours	present	conservative	improved
Yang *et al*. (2011) [24]	PSF	LS	56, F	21 hours	unknown	surgery	ataxia and aphasia
Hempelmann and Mater (2012) [10]	IETR	TS	61, F	7 days	present	conservative	improved
	PSF	LS	69, F	2 days	present	conservative	improved
	PSF	LS	62, F	1 day	present	conservative	improved
Khalatbari *et al*. (2012) [13]	discectomy	LS	53, M	8 hours	present	surgery	improved
	laminectomy	LS	75, M	perioperative	present	surgery	died
Lee *et al*. (2012) [16]	PSF	LS	63, F	6 hours	present	conservative	improved
Takahashi *et al*. (2012) [21]	laminoplasty	CS	69, F	15 hours	unknown	conservative	improved
Kaloostian *et al*. (2013) [11]	PSF	CS	45, M	perioperative	present	conservative	improved
	PSF	LS	64, F	2 days	present	conservative	brain dead
	PSF	LS	81, F	1 day	present	surgery	died
Yoo *et al*. (2013) [25]	intradural disc surgery	LS	66, M	2 days	present	surgery	improved

CS, Cervical spine; F, Female; IETR, Intradural extramedullary tumor resection; LS, Lumbar spine; M, Male; PSF, Posterior spinal fusion; TS, Thoracic spine.

RCH occurs in patients with a dural tear and CSF leakage, whether occult or not. It is thus believed that perioperative and/or postoperative CSF losses, leading to cranial hypotension, represent the main contributing factor in RCH [1,8,12]. The exact pathophysiology of RCH is still controversial. It is suggested that transient stretching and occlusion of superior cerebellar veins, resulting from downward cerebellar displacement under conditions of intracranial hypotension, may lead to cerebellar hemorrhagic infarction [8,20]. It is also suggested that cerebellar sag can directly cause tearing and bleeding of superior cerebellar veins [8]. Pallud *et al*. [20] hypothesized that RCH results primarily from superior cerebellar venous stretching and tearing, and that cerebellar infarction and swelling occur secondarily.

The loss of CSF should be restricted and controlled, because intracranial hypotension may be the initial cause of RCH. Closed wound suction drainage is recommended for spinal surgery, because a postoperative drain theoretically reduces the risk of infection and/or wound breakdown by decompressing the site of postoperative hematoma formation. However, if too much serosanguineous fluid drains postoperatively, stopping drainage or removing the drainage tube should be considered to prevent intracranial hypotension. Removal of the drain restores the normal CSF flow dynamics, allowing the cerebellum to resume its normal position [1]. Friedman *et al*. [8] described a 56-year-old woman with postoperative RCH whose headache resolved when suction drainage of the wound was discontinued. Thus, considering our case and the published literature, we suggest stopping drainage when RCH is suspected based on the patient’s complaints, including nausea and headache, and/or if an excessive amount of serosanguineous fluid has been drained postoperatively. This complication can be prevented by observing the amount of drainage fluid. If an excessive amount of fluid is drained, drainage should be stopped or converted to a gravity drain instead of a suction drain.

## Conclusions

RCH is a rare postoperative complication of spinal surgery. RCH must be suspected when intracranial symptoms or unexpected neurological signs occur after spinal surgery. If an excessive amount of serosanguineous fluid is found coming from the drainage tube postoperatively, drainage should be stopped.

## Consent

Written informed consent was obtained from the patient for publication of this case report and accompanying images. A copy of the written consent is available for review by the Editor-in-Chief of this journal.

## Abbreviations

CT: Computed tomography
CSF: Cerebrospinal fluid
CT: Computed tomography
MEP: Motor-evoked potential
MRI: Magnetic resonance imaging
RCH: Remote cerebellar hemorrhage
